# Residential exposure to natural outdoor environments and general health among older adults in Shanghai, China

**DOI:** 10.1186/s12939-019-1081-4

**Published:** 2019-11-21

**Authors:** Baishi Huang, Ye Liu, Zhiqiang Feng, Jamie R. Pearce, Ruoyu Wang, Yina Zhang, Jie Chen

**Affiliations:** 1 School of Geography and Planning, Sun Yat-Sen University, Guangzhou, 510275 China; 2 Guangdong Key Laboratory for Urbanization and Geo-simulation, Sun Yat-Sen University, Guangzhou, China; 3 School of GeoSciences, University of Edinburgh, Edinburgh, UK; 4 School of Social Development and Public Policy, Fudan University, Shanghai, China; 5 School of International and Public Affairs, Shanghai Jiao Tong University, Shanghai, China

**Keywords:** Green spaces, Blue spaces, Natural outdoor environment, Self-rated health, Older adults, Population census

## Abstract

**Background:**

Exposure to natural outdoor environments (NOE) has been shown to be beneficial to older adults’ health and functioning, yet this assertion has rarely been tested in China. We investigated the relationships between exposure to NOE and older adults’ self-rated health in Shanghai, China and examined whether these relationships varied by sex, age, education and hukou status.

**Method:**

This cross-sectional study used micro-data sample of the 2010 Shanghai population census, including 7962 older adults nested within 3345 neighbourhoods. Self-rated health was the outcome variable. Four NOE exposure indicators were calculated for each neighbourhood: the amount of surrounding greenness/blueness and proximity to large green/blue spaces. Multilevel logistic regression was employed to explore the association between natural outdoor environment exposure and self-rated health, adjusting for individual-level and neighbourhood-level covariates. Stratified analyses were used to examine variations by sex, age, education and hukou status.

**Results:**

Older adults living in neighbourhoods with higher surrounding greenness and higher proximity to both green spaces and blue spaces were more likely to report good health. Residential surrounding blueness was not significantly related to self-rated health. Females, those aged 60–69 years, those who had elementary school or junior high school education and those with non-local hukou benefit more from residential surrounding greenness, and those aged 70–79 years and who had elementary school or junior high school education benefit more from residential proximity to blue spaces.

**Conclusions:**

Higher residential greenness and proximity to both green spaces and blue spaces were associated with better self-rated health, particularly for females, younger older adults, the low educated and non-local hukou holders. Our findings suggest that urban green spaces and urban blue spaces have different effects on health among Chinese older adults and that the assessment of exposure matters to the investigation of NOE-health relationships.

## Introduction

China has the largest number of older people in the world and is currently experiencing rapid population aging. In 2010, China’s population over 60 years reached approximately 177.6 million, which accounted for 13.26% of the total population and is expected to reach 25% by 2030 [1]. Older people tend to be more dependent on the immediate residential neighbourhood environment due to declines in physical and cognitive functioning, reduction in social networks and increased fragility, and therefore their health is more closely entwined with their immediate residential environment [2]. Further, with rapid urbanization in China, urban dwellers have faced increased exposure to environmental hazards (e.g. air pollution, noise, extreme temperatures) and place-based factors (e.g. cigarette retailers) that undermine healthy lifestyles [3–6]. Identifying environmental attributes that support health and wellbeing amongst older populations in China is therefore a critical priority for policymakers.

A growing body of epidemiological evidence has shown that exposure to natural outdoor environments (NOE) is beneficial to older adults’ health and functioning [2]. NOE is associated with better self-reported mental health [7], reduced level of stress and mental disorders [8, 9] and reduction in all-cause mortality [10]. It is also positively associated with longevity [11], improved cardiovascular health [12] and increased levels of walking activity [13] among older adults. Some scholars have suggested that NOE in the vicinity of people’s homes (e.g. green spaces and blue spaces) provide a protective effect on health and wellbeing through multiple pathways including reducing exposure to environmental stressors, restoring attention, encouraging physical activity and facilitating neighbourhood social cohesion [10, 14–18].

A small but growing body of research has explored the salutogenic effects of residential surrounding greenness in China [10, 19–25], but relatively little attention has been paid to the salutogenic effects of blue spaces [26, 27]. Studies from developed countries have shown that large blue spaces provide accessible and attractive places for people to socialize with neighbours and conduct physical activity, with benefits for local health [28, 29]. However, empirical studies in Chinese rapidly urbanizing and densely populated cities are scant, and it is unclear whether experiences drawn from low-density cities in Western countries can be applied to Asian high-density settings. Although two studies have investigated the association between exposure to blue spaces and health among Chinese urban residents [26, 27], these studies are based on small-scale survey data collected in some places in a Chinese city (48 neighbourhoods in Beijing and one community-based health centre Hong Kong), which suffers from the problem of limited statistical power and poor generalizability. Furthermore, some earlier studies have suggested that NOE-health relationships varied by individual demographic and socioeconomic characteristics (e.g. age, sex, education and cultural background) [29–31] and that older people tend to benefit more from residential proximity to NOE than the younger [16]. But no research has examined the variation of NOE-health relationships by demographic and socioeconomic characteristics in China.

Therefore, this study investigated the relationships between exposure to NOE in the vicinity of homes (measured by the amount of surrounding greenness / blueness and the residential proximity to large green / blue spaces) and self-rated health (SRH) among older adults in Shanghai, a Chinese high-density megacity. We further examined whether these relationships differed by demographic and socioeconomic attributes such as sex, age, education and hukou status. This study is among the first to use large-scale representative data across an entire city to explore the relationships between NOE exposure and SRH among older people in Chinese cities. It uses multiple indicators (surrounding greenness/blueness and proximity to green spaces/blue spaces) to assess residents’ exposure to NOE, thus being able to capture the complexity of the salutogenic effects of NOE.

## Methods

### Study population

The focus for this study is Shanghai, which covers an area of 6340.5 km^2^ and has a total population of 23 million (the largest city in China by population). It accommodated 3.5 million population aged 60 or above, which accounted for 15.07% of the total population in 2010. The data used in our analysis were taken from the micro-data sample of the 2010 Shanghai population census together with high-resolution land cover datasets. The micro-data sample was randomly extracted from the 2010 Shanghai population census database using a systematic sampling technology, and the demographics of micro-data sample were representative of the Shanghai population. The micro-data sample contains 55,169 individuals living in 4895 neighbourhoods. The mean population of Shanghai’s neighbourhoods is 6465, and the mean size is 1.2 km^2^. We selected people aged 60 or above only for this study, because those aged under 60 years did not provide health information in the census. Among those aged 60 or above, 461 (5.4% of the total) individuals were omitted due to missing values for key variables. The neighbourhood in this study is defined as an area administered by neighbourhood committee (*Juweihui*), which is the smallest administrative division in urban China. The final dataset comprised 7962 older adults from 3354 neighbourhoods. Additional file 1: Figure S1 illustrates the process of selecting study samples. For authors from the Chinese institutions, the population census is exempt from ethical approval, since data used for the present study were anonymized and without any sensitive information. For authors from the United Kingdom institution, ethical approval for the study was obtained from the Edinburgh University School of Geosciences Research Ethics Committee.

### Outcome

Our outcome variable was SRH, which is a commonly used indicator to assess individual’s current health status [32, 33]. Respondents of the 2010 Shanghai population census were asked “In general, how would you rate your health over the past month?” They could respond with one of the following four categories: ‘good health’, ‘fair health’, ‘poor health’ and ‘not able to take care of myself’. We removed respondents who answered ‘not able to take care of myself’ (*n* = 305), because they were mostly advanced aging people above 80 years old (*n* = 180) who had a higher risk of mortality. We then dichotomized other categories into ‘good health’ and ‘fair or poor health’ (as the referenced category).

### Exposure to natural outdoor environments

Exposure to NOE were derived from GlobeLand30–2010 with 30 m × 30 m spatial resolution. The GlobeLand30–2010 were extracted from Landsat and Chinese HJ-1 satellite images around 2010. Land cover was classified into ten types, including cultivated land, forest, grassland, shrubland, wetland, water bodies, tundra, artificial surfaces, bareland, permanent snow and ice [34]. We grouped cultivated land, forest, grassland, shrubland and wetland into green spaces and defined blue spaces as water bodies. Following previous studies, we used two sets of indicators to assess individuals’ exposure to NOE [15]: the amount of surrounding greenness/blueness and proximity to green space/blue space. “Surrounding area” is defined as a circular buffer with a specific radius around the centroid of participants’ residential neighbourhood in the sense of an administrative division, and “proximity” is defined as the straight-line distance between neighbourhood centroid to the boundary of the nearest major NOE. Specifically, we developed the following four NOE exposure indicators: 1) the amount of surrounding greenness, which was measured by the total percentage of green spaces within a circular buffer with a radius of 1 km around the centroid of participant’s residential neighbourhood, 2) the amount of surrounding blueness, which was assessed by the proportion of blue spaces within the same circular buffer, 3) proximity to large green spaces, measured as the straight line distance between the centroid of respondent’s residential neighbourhood to the boundary of the nearest green spaces larger than 1 ha, 4) proximity to large blue spaces, assessed using the straight line distance between the centroid of respondent’s neighbourhood and the boundary of the nearest blue spaces larger than 1 ha. We treated the amount of surrounding greenness / blueness and proximity to large green / blue spaces as continuous variables. A larger value of surrounding greenness / blueness represented a greater amount of surrounding greenness / blueness, and a smaller value of proximity to major green / blue spaces represented closer proximity to major green / blue spaces.

### Covariates

We used neighbourhood social deprivation index to capture neighbourhood socioeconomic status [35]. Following Townsend [36] and Carstairs et al. [37], we created neighbourhood social deprivation index based on four neighbourhood-level indicators from the 2010 Shanghai population census: unemployment rates, the proportion of residents with junior high school education or below, the proportion of residents working in the low-end occupations (including employees in commerce and service sectors, employees in farming, forestry, animal husbandry and fishery sectors, and people operating the manufacturing and transportation equipment and related personnel) and the proportion of residents who are tenants. A higher index indicates greater levels of neighbourhood-level social deprivation.

Air pollution, especially fine particulate matter, has been regarded as a potential confounder in NOE-health relationships [8, 17, 38]. This study includes annual average PM2.5 concentrations as a covariate, assuming that salutogenic impacts of green spaces might be over-estimated due to lower levels of air pollution. PM2.5 data (provided at a spatial resolution of 0.01 degree × 0.01 degree in 2010) were obtained from Socioeconomic Data and Applications Centre website (http://sedac.ciesin.columbia.edu/data/set/sdei-global-annual-gwr-pm2-5-modis-misr-seawifs-aod/data-download) [39] and were used to generate neighbourhood-level index of PM2.5 concentrations using QGIS 3.6 software.

Additional individual-level confounding covariates were adjusted for: age (60–69 vs 70–79 vs > =80), sex (male vs female), marital status (single, divorced, or widowed vs married), hukou status (governmental household registration system, a marker of socioeconomic status and cultural background, local hukou vs non-local hukou), education (no schooling vs elementary school or junior high school vs senior high school vs college or above), living alone (yes vs no), housing area per capita (continuous variable), housing construction time (before 1980 vs after 1980, a proxy of household economic status), and housing facilities (including water supply, kitchen, toilet and bathroom) (none, one, two or three types of facilities vs four types of facilities) [10, 18, 27]. All individual-level covariates were obtained from the micro-data sample of the 2010 Shanghai population census.

### Statistical analysis

The relationships between SRH and NOE exposure were examined using multilevel logistic regressions. The baseline model (Model 1) included neighbourhood-level and individual-level covariates only, namely, social deprivation index, annual average PM2.5 concentration, age, sex, marital status, hukou status, education, living alone, housing area per capita, housing construction time and housing facilities. We then added predictors of the amount of surrounding greenness/blueness (Model 2) and access to green space/blue space (Model 3) separately to the baseline model. We further investigated potential variation in NOE-health relationships across strata of sex, age, education and hukou status using stratified analyses. Sex and age are basic demographic attributes, and education and hukou status are commonly used to measure socioeconomic status in China. Finally, we conducted several sensitivity analyses: 1) we changed the radius of buffer areas from 1 km to 2 or 3 km and then reran the regressions, 2) we changed the minimum size of green space/blue space from 1 ha to 0.5 ha and then reran the regressions, 3) we categorised continuous variables of exposure to NOE into tertiles. We found no evidence of multicollinearity among the variables by performing a variance inflation factor test. Analyses were conducted using Stata 14.0.

## Results

Table 1 shows the descriptive statistics of the sample. Among 7962 older adults, 3585 (45.0%) participants reported good health, 4377 (55.0%) reported fair or poor health. Respondents were more likely to be female, younger, married, holding local hukou, junior high school education or below, not living alone, individuals who lived in houses constructed after 1980 and in houses equipped with four types of facilities. The average housing area per capita was 29.2 square meters. Participants who reported good health tended to reside in neighbourhoods with higher surrounding greenness/blueness and closer proximity to green space/blue space. There were significant differences in demographic and socioeconomic characteristics between those who reported good health and those who reported fair or poor health.

**Table 1:** Summary statistics of variables

Variables	Whole sample (*n* = 7962)	Reported good health (*n* = 3585)	Reported fair or poor health (*n* = 4377)	*p*-value
Outcome				
Self-reported health (%)				
Good	45.03			
Fair or poor	54.97			
Predictors (neighbourhood-level variables)				
Percentage of surrounding green spaces within 1 km buffer (%)	16.41 (27.67)	18.27 (28.38)	14.88 (26.98)	0.000^a^
Percentage of surrounding blue spaces within 1 km buffer (%)	1.76 (5.61)	1.84 (5.75)	1.69 (5.50)	0.227^a^
Proximity to the nearest green spaces (metre)	775.98 (702.45)	715.42 (698.82)	825.59 (701.60)	0.000^a^
Proximity to the nearest blue spaces (metre)	2108.24 (1512.03)	2081.94 (1498.20)	2129.79 (1523.09)	0.160^a^
Covariates				
Social deprivation index	−0.31 (1.91)	−0.27 (2.02)	−0.34 (1.80)	0.146^a^
Annual average PM2.5 concentrations (μg/m^3^)	51.81 (2.59)	51.83 (3.17)	51.80 (1.98)	0.596^a^
Sex (%)				
Male	48.47	53.22	44.57	0.000^b^
Female	51.53	46.78	55.43	
Age (years) (%)				
60–69	53.49	70.88	39.25	0.000^b^
70–79	30.75	22.51	37.49	
> =80	15.76	6.61	23.26	
Marital status (%)				
Single, divorced, or widowed	22.70	14.64	29.29	0.000^b^
Married	77.30	85.36	70.71	
Hukou status (%)				
Local hukou	68.12	63.57	71.85	0.000^b^
Non-local hukou	31.88	36.43	28.15	
Education (%)				
No schooling	14.46	10.38	17.80	0.000^b^
Elementary school or junior high school	55.15	58.02	52.80	
Senior high school	16.68	17.10	16.34	
College or above	13.71	14.50	13.06	
Living alone (%)				
Yes	6.66	5.38	7.70	0.000^b^
No	93.34	94.62	92.30	
Housing area per capita (m^2^)	29.17 (22.83)	30.82 (24.03)	27.84 (21.70)	0.000^a^
Housing construction time (%)				
Before 1980	15.02	11.58	17.84	0.000^b^
After 1980	84.98	88.42	82.16	
Housing facilities (%)				
None, one, two or three types of facilities	13.26	11.83	85.56	0.001^b^
Four types of facilities	86.74	88.17	14.44	

Note: results are presented as proportion for categorical variables and as mean (standard errors) for continuous variables

^a^t-Test statistics. ^b^Pearson’s chi-squares statistics

Table 2 shows results from the multilevel logistic regression. The between-neighbourhood variance and neighbourhood-level intra-class correlation of the null model were 3.53 and 0.52, respectively, which indicates the validity of the multilevel model. Model 1 includes covariates only. Individuals who were males and held non-local hukou were more likely than females and local hukou holders to report good health. As anticipated, individuals who were older, who were not married and who lived in more recently constructed houses were less likely than their younger, married and older-house counterparts to report good health. However, no significant relationship was found between SRH and social deprivation and between SRH and annual average PM2.5 concentrations.

**Table 2:** Multilevel logistic regression estimates of reporting good health

Effects and variables	Model 1^a^	Model 2^b^	Model 3^c^
	OR (95% CI)	OR (95% CI)	OR (95% CI)
Fixed variables			
The percentage of surrounding green spaces within 1 km buffer		1.75 (1.15–2.65) **	
The percentage of surrounding blue spaces within 1 km buffer		2.54 (0.49–13.12)	
Logarithm of proximity to the nearest green spaces			0.93 (0.89–0.97) **
Logarithm of proximity to the nearest blue spaces			0.90 (0.84–0.98) *
Social deprivation index	1.05 (0.99–1.10)	1.01 (0.95–1.07)	1.00 (0.94–1.06)
Annual average PM2.5 concentrations	1.00 (0.97–1.04)	1.00 (0.97–1.04)	1.00 (0.97–1.03)
Males (ref: females)	1.50 (1.31–1.71) ***	1.48 (1.30–1.70) ***	1.48 (1.30–1.70) ***
Age (ref: 60–69)			
70–79	0.21 (0.17–0.25) ***	0.21 (0.18–0.25) ***	0.21 (0.18–0.25) ***
> =80	0.08 (0.06–0.11) ***	0.08 (0.06–0.11) ***	0.08 (0.06–0.11) ***
Single, divorced, or widowed (ref: married)	0.65 (0.53–0.80) ***	0.65 (0.53–0.80) ***	0.65 (0.53–0.80) ***
Living alone (ref: no)	0.88 (0.63–1.23)	0.91 (0.65–1.28)	0.91 (0.65–1.28)
Education (ref: no schooling)			
Elementary school or junior high school	1.04 (0.83–1.31)	1.10 (0.87–1.39)	1.11 (0.88–1.40)
Senior high school	0.91 (0.68–1.22)	0.99 (0.74–1.33)	1.00 (0.75–1.34)
College or above	1.01 (0.74–1.39)	1.10 (0.80–1.52)	1.12 (0.82–1.54)
Non-local hukou (ref: local hukou)	1.41 (1.20–1.67) ***	1.44 (1.22–1.71) ***	1.44 (1.22–1.70) ***
Housing area per capita	1.00 (0.99–1.01)	1.00 (0.99–1.01)	1.00 (1.00–1.01)
Housing construction time after 1980 (ref: before 1980)	0.69 (0.53–0.90) **	0.72 (0.55–0.94) *	0.73 (0.56–0.95) *
Housing facilities (ref: none, one, two and three)	1.09 (0.84–1.43)	1.04 (0.79–1.36)	1.03 (0.78–1.35)
Random variables			
Var (neighbourhood-level constant)	3.82 (3.23–4.52) ***	3.81 (3.22–4.50) ***	3.80 (3.22–4.49) ***
ICC	0.54	0.54	0.54
Number of neighbourhoods	3354	3354	3354
Number of individuals	7962	7962	7962
AIC	9273.95	9269.76	9260.74

Note: ^a^Model 1 included social deprivation index, annual average PM2.5 concentration, age, sex, marital status, hukou status, education, living alone, housing area per capita, housing construction time and housing facilities

^b^Model 2 included variables in Model 1, the percentage of surrounding green spaces within 1 km buffer and the percentage of surrounding blue spaces within 1 km buffer to Model 1

^c^Model 3 included variables in Model 1 and the logarithm of proximity to the nearest green spaces and the logarithm of proximity to the nearest blue spaces to Model 1

OR odds ratio; 95% confidence intervals in brackets; * *p* < 0.05, ** *p* < 0.01, *** *p* < 0.001

The results of Model 2 show that the percentage of surrounding green spaces within 1 km buffer was positively associated with the likelihood of reporting good health (OR = 1.75, 95%CI 1.15 to 2.65), while the percentage of surrounding blue spaces within 1 km buffer was not significantly associated with the odds of reporting good health (OR = 2.54, 95%CI 0.49 to 13.12). This suggested that older people who lived in neighbourhoods with higher surrounding greenness (but not blueness) had a better SRH. Model 3 shows that proximity to both the nearest green spaces and the nearest blue spaces with a minimum size of 1 ha were negatively related to the odds of reporting good health (OR = 0.93, 95%CI 0.89 to 0.97 and 0.90, 95%CI 0.84 to 0.98, respectively). This indicated that older adults who lived in neighbourhoods with better access to green space/blue space were more likely to report good health. Results from sensitively analyses confirm the robustness of results (Additional file 1: Tables S1-S3).

Table 3 shows the results of stratified analyses. For sex-stratified analyses, the percentage of and accessibility to green spaces were associated with SRH among females (OR = 1.64, 95%CI 1.14 to 2.37 and OR = 0.94, 95%CI 0.91 to 0.98, respectively), while the association between proximity to the nearest blue spaces and SRH were similar for females and males (OR = 0.94, 95%CI 0.88 to 0.99 and OR = 0.94, 95%CI 0.89 to 0.99, respectively). For age-stratified analyses, the amount of surrounding greenness and proximity to green space were more strongly linked to SRH among older adults aged 60–69 years (OR = 2.39, 95%CI 1.27 to 4.49 and OR = 0.91, 95%CI 0.85 to 0.97, respectively). However, for proximity to blue space, its association with SRH was stronger and attained statistical significance only for those aged 70–79 years (OR = 0.84, 95%CI 0.72 to 0.97). For education-stratified analyses, the amount of surrounding greenness, proximity to both green spaces and blue spaces were associated with SRH among those who had elementary school or junior high school education (OR = 2.09, 95%CI 1.22 to 3.59, OR = 0.92, 95%CI 0.87 to 0.98 and OR = 0.85, 95%CI 0.77 to 0.94, respectively). For hukou status stratified analyses, we observed stronger protective effects of the amount of surrounding greenness and residential proximity to green space on older adults’ SRH among respondents who did not have local hukou (OR = 4.26, 95%CI 1.51 to 12.01 and OR = 0.85, 95%CI 0.78 to 0.94, respectively).

**Table 3:** Association between natural outdoor environments and the odds of reporting good health

Sex	Females	Males		
	OR (95% CI)	OR (95% CI)		
The percentage of surrounding green spaces within 1 km buffer	1.64 (1.14–2.37) **	1.32 (0.94–1.86)		
The percentage of surrounding blue spaces within 1 km buffer	3.10 (0.75–12.88)	1.14 (0.31–4.19)		
Logarithm of proximity to the nearest green spaces	0.94 (0.91–0.98) **	0.97 (0.94–1.00)		
Logarithm of proximity to the nearest blue spaces	0.94 (0.88–0.99) *	0.94 (0.89–0.99) *		
Age (years)	60–69	70–79	> = 80	
	OR (95% CI)	OR (95% CI)	OR (95% CI)	
The percentage of surrounding green spaces within 1 km buffer	2.39 (1.27–4.49) **	1.48 (0.66–3.30)	1.01 (0.23–4.35)	
The percentage of surrounding blue spaces within 1 km buffer	2.80 (0.24–32.39)	3.21 (0.13–82.10)	1.91 (0.01–269.42)	
Logarithm of proximity to the nearest green spaces	0.91 (0.85–0.97) **	0.95 (0.87–1.03)	0.94 (0.81–1.09)	
Logarithm of proximity to the nearest blue spaces	0.92 (0.82–1.03)	0.84 (0.72–0.97) *	1.00 (0.78–1.28)	
Education	No schooling	Elementary school or junior high school	Senior high school	College or above
	OR (95% CI)	OR (95% CI)	OR (95% CI)	OR (95% CI)
The percentage of surrounding green spaces within 1 km buffer	1.66 (0.70–3.92)	2.09 (1.22–3.59) **	3.44 (0.71–16.60)	2.18 (0.14–33.14)
The percentage of surrounding blue spaces within 1 km buffer	8.31 (0.35–198.67)	7.17 (0.81–63.61)	0.03 (0.00–2.28)	0.03 (0.00–143.17)
Logarithm of proximity to the nearest green spaces	0.96 (0.87–1.05)	0.92 (0.87–0.98) **	0.89 (0.79–1.00)	0.86 (0.70–1.06)
Logarithm of proximity to the nearest blue spaces	0.90 (0.77–1.06)	0.85 (0.77–0.94) **	0.99 (0.83–1.17)	1.09 (0.80–1.49)
Hukou status	Local hukou	Non-local hukou		
	OR (95% CI)	OR (95% CI)		
The percentage of surrounding green spaces within 1 km buffer	1.53 (0.91–2.57)	4.26 (1.51–12.01) **		
The percentage of surrounding blue spaces within 1 km buffer	3.85 (0.54–27.67)	1.06 (0.18–61.40)		
Logarithm of proximity to the nearest green spaces	0.95 (0.90–1.01)	0.85 (0.78–0.94) **		
Logarithm of proximity to the nearest blue spaces	0.90 (0.82–0.99)	0.88 (0.75–1.04)		

Note: OR odds ratio; 95% confidence intervals in brackets; * *p* < 0.05, ** *p* < 0.01, *** *p* < 0.001

All models have been adjusted for all covariates shown in Table 2

## Discussion

This study examined the relationships between NOE exposure and SRH among older adults in Shanghai, China, and explored whether these relationships varied by sex, age, education and hukou status. We found that older adults living in neighbourhoods with higher surrounding greenness were more likely to report good health, and their proximity to both green spaces and blue spaces was associated with better SRH. However, no significant association between residential surrounding blueness and SRH was observed. Stratified analyses suggested more beneficial effects of residential surrounding greenness for females, those aged 60–69 years, those who had elementary school or junior high school education and non-local hukou holders and more beneficial effect of access to blue spaces for those aged 70–79 years and those who had elementary school or junior high school education.

Our findings of a positive association between residential greenness exposure and SRH are consistent with findings from previous studies conducted in developed countries [14, 16, 29, 30, 35, 40]. However, insufficient attention has been devoted to the association among older adults living in China’s megacity. We found a higher odds of reporting good health for those who lived in neighbourhoods with higher surrounding greenness and closer proximity to green spaces. This is in contrast to some previous studies [12, 14, 29], which showed a positive linkage between residential surrounding greenness and health but no correlation between the presence of major green spaces within a buffer and health. This discrepancy may be due to different study populations, different measures of proximity to green spaces and different environmental context. Compared to working-age population, older population are less mobile and are more dependent on surrounding green spaces for social life, recreation and leisure. This is particularly the case for Shanghai’s older people, who tend to travel by public transport with short distances owing to decreased mobility [41, 42]. A similar study carried out in Barcelona found that subjective proximity to green spaces was associated with better general health but no association between objective proximity to green spaces and health [14]. Another reason for the discrepancy in results is using different measures of proximity to green spaces. The current study uses straight-line distance, instead of the presence of major green spaces in the vicinity of respondents’ homes [9, 12, 14, 29] or road-network distance [43–45], as a proxy of proximity to green spaces. When the presence of major green spaces around the residential address was used as the proxy, no statistically significant relationship was found with general health as well as the prevalence of non-communicable diseases in Spain and Lithuania [12, 14]. When different types of buffers (e.g. circular buffer, network buffer, and nested buffer) and different metrics of green spaces (e.g. vegetation cover, canopy cover, and park area) were used, the association between self-reported mental health and exposure to urban green spaces turned out to vary in Singapore [46].

Residential surrounding blueness was found to be not related to older adults’ SRH, whereas residential proximity to major blue spaces was positively linked to better SRH. These results hint at the importance of large blue spaces (e.g. rivers and lakes) in providing accessible and attractive settings for nearby older people to socialize with their neighbours and engage in physical activity. The insignificant association between the amount of surrounding blueness and SRH may be attributable to the small between-neighbourhood variation of the percentage of surrounding blue spaces. For example, the descriptive statistics show that 90% neighbourhoods in Shanghai have lower than 5% blue spaces as land cover within a 1 km buffer around the neighbourhood centroid, and more than half neighbourhoods do not have any water body within the buffer. By contrast, the variation of residential proximity to large blue spaces is much larger, which is possible for researchers to detect the salutogenic effects of blue spaces. The existing evidence on the beneficial effect of blue spaces on health is still inconclusive. Although a few studies showed that exposure to blue spaces was related to better health [26, 47], other studies did not lead to the same conclusion [9, 27, 29]. Further empirical research is needed to offer more conclusive evidence on the mechanisms underlying the health benefit of blue spaces [15].

In line with previous studies [12, 18, 29], we observed that beneficial effects of residential greenness exposure were stronger for females compared with males. Possible explanation for this variation is that older females are assumed to use green spaces more frequently and for a longer time than older males, as in the Chinese context, female retirees usually spend more time taking care of their grandchildren and socialising with their neighbours in neighbourhood open spaces than their male counterparts [12]. We also found that the linkages between green spaces exposures and SRH were stronger for those aged 60–69 years. This finding is in partial agreement with findings from a Dutch study that the relationship between green space and prevalence of physician-assessed morbidity was stronger for people aged between 46 and 65 years than older people (65+ years) [48]. One reason behind this relationship is that younger older people are more willing to and are more able to use green spaces in the vicinity of their homes for recreational and social activities than the advanced aged. Our findings of stronger associations between green spaces exposures and SRH for those with lower education attainment are consistent with previous studies [16–18, 31, 48]. Low-educated participants tend to spend more time near their home and thus are more influenced by their immediate neighbourhood environment and public facilities [18, 231]. However, older adults aged 70–79 years and who had elementary school or junior high school education appear to benefit more from residential proximity to major blue spaces than other age groups. The associations between exposures to green spaces and SRH are stronger for non-local hukou holders. This reflects the fact that non-local hukou holders normally travelled by public transport for a short distance rather than by car for a long distance, as they had lower income and less wealth than local hukou holders, and their social and leisure activity spaces were restricted to the vicinity of their homes [49].

We did not find any relationship between social deprivation index and older adults’ SRH and between annual average PM2.5 concentrations and their SRH. Previous studies reported a negative relationship between neighbourhood deprivation and health [50–52] and between air pollution and health [9, 17]. However, the inverse relationships were not observed in the current study. This finding can be partially attributable to differences in socio-cultural context, measurement used and the characteristics of participants. Another possible explanation is that there is a small variation in the level of social deprivation index and the level of annual average PM2.5 concentrations across Shanghai, which may lead to unexpected insignificant linkages.

This study has some limitations. First, the cross-sectional design is not able to prove a causal inference in relation to our explored significant associations. Second, our outcome variable is based on self-rated measure, which could lead to outcome misclassification. Further research is needed to verify our results by employing objective measures of health using other datasets. Third, our metric of neighbourhood NOE did not take into account respondents’ use of and satisfaction with NOE and the type and quality of NOE due to data limitation. Fourth, we used straight line distance to measure respondents’ proximity to green spaces/blue spaces, but it would be more accurate to use other metrics such as road network distance. Fifth, we were unable to adjust for some individual socioeconomic variables (e.g. income and employment) due to the unavailability of relevant information in our dataset. This may cause omitted-variable bias in regression estimates. Sixth, there is a possibility of bias in regression estimates due to neighbourhood selection. For example, older people who are socially active, autonomous and physically active are more likely to report better SRH than other older people, and the former group is more willing to live in neighbourhood with better access to green spaces than the latter group. In this case, omitting variables regarding older adults’ characters and personalities may lead to an overestimate in the relationship between residential proximity to green spaces and SRH.

## Conclusion

Residential surrounding greenness and residential proximity to green spaces were associated with better SRH among older adults in Shanghai, China. The association between proximity to blue spaces and better SRH was relatively small, and the association regarding surrounding blueness was not conclusive. Results from stratified analyses indicated that the relationships were stronger for females, those aged 60–69 years, those with elementary school or junior high school education and non-local hukou holders. Our findings provide epidemiological evidence that urban green spaces and urban blue spaces have different effects on health among Chinese older adults and that the assessment of exposure matters to the investigation of NOE-health relationships. Our findings suggest that exposure to NOE, especially urban green spaces, are associated with better SRH among older adults living in a high-density setting. Therefore, to achieve an age-friendly society and build a healthy city, urban planners and landscape designers are advised to develop innovative approaches (e.g. providing a small patch of green space after demolishing several old buildings) to develop green/blue infrastructure at the neighbourhood level to increase residents’ opportunities of contacting with NOE.

## Supplementary information

**Additional file 1: Figure S1.** The process of selecting study samples. **Table S1.** Multilevel logistic regression analysis for the association between percentage of green / blue spaces within 2 km and 3 km buffer and reporting good health. **Table S2**. Multilevel logistic regression analysis for the association between distance to the nearest green / blue spaces (larger than 0.5 ha and any size) and reporting good health. **Table S3.** Multilevel logistic regression analysis for the association between NOE exposure measured as tertiles and reporting good health.

## Data Availability

The datasets used and/or analysed during the current study are available from the corresponding author on reasonable request.

## Abbreviations

AIC: Akaike information criterion
CI: Confidence interval
NOE: Natural outdoor environment
OR: Odds ratio
PM2.5: Fine particulate matter
SRH: Self-rated health
