# Supplementary material for: Residential exposure to natural outdoor environments and general health among older adults in Shanghai, China
Source: Int J Equity Health. 2019 Nov 21;18:178. doi: 10.1186/s12939-019-1081-4 (PMC6868833; doi:10.1186/s12939-019-1081-4)
Supplement: Supplementary file 1 — Additional file 1: Figure S1. The process of selecting study samples. Table S1. Multilevel logistic regression analysis for the association between percentage of green / blue spaces within 2 km and 3 km buffer and reporting good health. Table S2. Multilevel logistic regression analysis for the association between distance to the nearest green / blue spaces (larger than 0.5 ha and any size) and reporting good health. Table S3. Multilevel logistic regression analysis for the association between NOE exposure measured as tertiles and reporting good health. [file 12939_2019_1081_MOESM1_ESM.docx]

**Residential exposure to natural outdoor environments and general health among older adults in Shanghai, China**

Baishi Huang^1,2^, Ye Liu^1,2*^, Zhiqiang Feng^3^, Jamie R Pearce^3^, Ruoyu Wang^3^, Yina Zhang^4^, Jie Chen^5*^

Figure S1. The process of selecting study samples

**Table S1 Multilevel logistic regression analysis for the association between percentage of green / blue spaces within 2 km and 3 km buffer and reporting good health**

| **Effects and variables** | **Model 4 (IV: within 2 km buffer)** | **Model 5 (IV: within 3 km buffer)** |
| --- | --- | --- |
|  | **OR (95% CI)** | **OR (95% CI)** |
| Fixed part |  |  |
| Percentage of green spaces | 2.00 (1.29 - 3.12) ** | 2.17 (1.38 - 3.39) *** |
| Percentage of blue spaces | 1.82 (0.38 - 8.75) | 1.00 (0.23 - 4.22) |
| Social deprivation index | 1.00 (0.95 - 1.06) | 1.00 (0.94 - 1.06) |
| Annual average PM2.5 concentrations | 1.00 (0.97 - 1.04) | 1.00 (0.97 - 1.04) |
| Males (ref: females) | 1.48 (1.29 - 1.70) *** | 1.48 (1.29 - 1.69) *** |
| Age (ref: 60-69) | |  |
| 70-79 | 0.21 (0.18 - 0.25) *** | 0.21 (0.18 - 0.25) *** |
| >=80 | 0.08 (0.06 - 0.11) *** | 0.08 (0.06 - 0.11) *** |
| Single, divorced, or widowed (ref: married) | 0.66 (0.53 - 0.80) *** | 0.66 (0.53 - 0.81) *** |
| Living alone (ref: no) | 0.92 (0.65 - 1.28) | 0.92 (0.65 - 1.29) |
| Education (ref: no schooling) | |  |
| Elementary school or junior high school | 1.10 (0.87 - 1.40) | 1.10 (0.87 - 1.39) |
| Senior high school | 1.00 (0.75 - 1.34) | 1.00 (0.75 - 1.35) |
| College or above | 1.12 (0.81 - 1.54) | 1.12 (0.81 - 1.54) |
| Non-local hukou (ref: local hukou) | 1.44 (1.22 - 1.70) *** | 1.43 (1.21 - 1.69) *** |
| Housing area per capita | 1.00 (1.00 - 1.01) | 1.00 (1.00 - 1.01) |
| Housing construction time after 1980 (ref: before 1980) | 0.73 (0.56 - 0.95) * | 0.73 (0.56 - 0.96) * |
| Housing facilities (ref: none, one, two and three) | 1.02 (0.78 - 1.35) | 1.03 (0.78 - 1.35) |
| Random part |  |  |
| Var (neighbourhood-level constant) | 3.81 (3.22 - 4.50) *** | 3.81 (3.23 - 4.51) *** |
| Number of neighbourhoods | 3354 | 3354 |
| Number of individuals | 7962 | 7962 |
| AIC | 9267.93 | 9266.44 |

Note: OR odds ratio; 95% confidence intervals in brackets; * p<0.05, ** p<0.01, *** p<0.001.

**Table S2** **Multilevel logistic regression analysis for the association between distance to the nearest green / blue spaces (larger than 0.5 hectare and any size) and reporting good health**

| **Effects and variables** | **Model 6 (IV: larger than 0.5 hectare)** | **Model 7 (IV: any size)** |
| --- | --- | --- |
|  | **OR (95% CI)** | **OR (95% CI)** |
| Fixed part |  |  |
| Logarithm of distance to the nearest green spaces | 0.93 (0.89 - 0.97) ** | 0.94 (0.90 - 0.98) ** |
| Logarithm of distance to the nearest blue spaces | 0.93 (0.86 - 1.00) # | 0.91 (0.85 - 0.99) * |
| Social deprivation index | 1.00 (0.95 - 1.06) | 1.00 (0.95 - 1.06) |
| Annual average PM2.5 concentrations | 1.00 (0.97 - 1.03) | 1.00 (0.97 - 1.03) |
| Males (ref: females) | 1.48 (1.29 - 1.70) *** | 1.48 (1.29 - 1.70) *** |
| Age (ref: 60-69) | |  |
| 70-79 | 0.21 (0.18 - 0.25) *** | 0.21 (0.18 - 0.25) *** |
| >=80 | 0.08 (0.06 - 0.11) *** | 0.08 (0.06 - 0.11) *** |
| Single, divorced, or widowed (ref: married) | 0.65 (0.53 - 0.80) *** | 0.65 (0.53 - 0.80) *** |
| Living alone (ref: no) | 0.91 (0.65 - 1.28) | 0.91 (0.65 - 1.27) |
| Education (ref: no schooling) | |  |
| Elementary school or junior high school | 1.10 (0.87 - 1.38) | 1.10 (0.87 - 1.39) |
| Senior high school | 1.00 (0.74 - 1.32) | 1.00 (0.74 - 1.33) |
| College or above | 1.12 (0.80 - 1.52) | 1.12 (0.81 - 1.54) |
| Non-local hukou (ref: local hukou) | 1.44 (1.21 - 1.70) *** | 1.44 (1.22 - 1.70) *** |
| Housing area per capita | 1.00 (0.99 - 1.01) | 1.00 (1.00 - 1.01) |
| Housing construction time after 1980 (ref: before 1980) | 0.73 (0.57 - 0.96) * | 0.74 (0.56 - 0.96) * |
| Housing facilities (ref: none, one, two and three) | 1.03 (0.79 - 1.37) | 1.03 (0.79 - 1.36) |
| Random part |  |  |
| Var (neighbourhood-level constant) | 3.81 (3.22 - 4.50) *** | 3.80 (3.22 - 4.49) *** |
| Number of neighbourhoods | 3354 | 3354 |
| Number of individuals | 7962 | 7962 |
| AIC | 9265.02 | 9261.74 |

Note: OR odds ratio; 95% confidence intervals in brackets; # p=0.054, * p<0.05, ** p<0.01, *** p<0.001.

**Table S3 Multilevel logistic regression analysis for the association between NOE exposure measured as tertiles and reporting good health**

| **Effects and variables** | **Model 8** | **Model 8** |
| --- | --- | --- |
|  | **OR (95% CI)** | **OR (95% CI)** |
| Fixed part |  |  |
| The percentage of green spaces within 1 km buffer (ref: 1st tertile) | |  |
| 2nd tertile | 1.14 (0.90 - 1.45) |  |
| 3rd tertile | 1.74 (1.33 - 2.26) *** |  |
| The percentage of blue spaces within 1 km buffer (ref: 1st tertile) | |  |
| 2nd tertile | 0.60 (0.28 - 1.30) |  |
| 3rd tertile | 1.12 (0.91 - 1.37) |  |
| Distance to the nearest green spaces (ref: 1st tertile) |  |  |
| 2nd tertile |  | 0.69 (0.53 - 0.89) ** |
| 3rd tertile |  | 0.59 (0.45 - 0.76) *** |
| Distance to the nearest blue spaces (ref: 1st tertile) |  |  |
| 2nd tertile |  | 0.83 (0.66 - 1.06) |
| 3rd tertile |  | 0.81 (0.64 - 1.03) # |
| Social deprivation index | 1.00 (0.95 - 1.06) | 1.00 (0.94 - 1.06) |
| Annual average PM2.5 concentrations | 1.00 (0.96 - 1.03) | 1.00 (0.96 - 1.03) |
| Males (ref: females) | 1.48 (1.29 - 1.69) *** | 1.48 (1.30 - 1.70) *** |
| Age (years) (ref: 60-69) |  |  |
| 70-79 | 0.21 (0.18 - 0.25) *** | 0.21 (0.18 - 0.25) *** |
| >=80 | 0.08 (0.06 - 0.11) *** | 0.08 (0.06 - 0.11) *** |
| Single, divorced, or widowed (ref: married) | 0.60 (0.53 - 0.81) *** | 0.65 (0.53 - 0.80) *** |
| Living alone (ref: no) | 0.92 (0.66 - 1.29) | 0.92 (0.66 - 1.29) |
| Education (ref: no schooling) |  |  |
| Elementary school or junior high school | 1.11 (0.88 - 1.40) | 1.10 (0.87 - 1.39) |
| Senior high school | 1.01 (0.76 - 1.35) | 1.00 (0.75 - 1.34) |
| College or above | 1.14 (0.83 - 1.57) | 1.12 (0.82 - 1.54) |
| Non-local hukou (ref: local hukou) | 1.42 (1.20 - 1.68) *** | 1.42 (1.20 - 1.67) *** |
| Housing area per capita | 1.00 (1.00 - 1.01) | 1.00 (1.00 - 1.01) |
| Housing construction time after 1980 (ref: before 1980) | 0.75 (0.58 - 0.98) * | 0.74 (0.57 - 0.97) * |
| Housing facilities (ref: none, one, two and three) | 1.04 (0.79 - 1.37) | 1.03 (0.79 - 1.35) |
| Random part |  |  |
| Var (neighbourhood-level constant) | 3.80 (3.21 - 4.49) *** | 3.79 (3.21 - 4.49) *** |
| Number of neighbourhoods | 3354 | 3354 |
| Number of individuals | 7962 | 7962 |
| AIC | 9260.49 | 9262.17 |

Note: OR odds ratio; 95% confidence intervals in brackets; # p=0.080, * p<0.05, ** p<0.01, *** p<0.001.
